# Molecular Characterization of Circulating Microbiome Signatures in Rheumatoid Arthritis

**DOI:** 10.3389/fcimb.2019.00440

**Published:** 2020-01-22

**Authors:** Dargham B. M. Hammad, S. L. Hider, Veranja C. Liyanapathirana, Daniel P. Tonge

**Affiliations:** ^1^Faculty of Natural Sciences, School of Life Sciences, Keele University, Keele, United Kingdom; ^2^Arthritis Research UK Primary Care Centre, Research Institute for Primary Care and Health Sciences, Keele University, Keele, United Kingdom; ^3^Haywood Academic Rheumatology Group, Midlands Partnership Foundation Trust, Staffordshire, United Kingdom; ^4^Department of Microbiology, Faculty of Medicine, University of Peradeniya, Peradeniya, Sri Lanka

**Keywords:** microbiome, rheumatoid, 16S, blood microbiome, biomarker

## Abstract

Rheumatoid Arthritis (RA) has been increasingly associated with perturbations to the microbial communities that reside in and on the body (the microbiome), in both human and animal studies. To date, such studies have mainly focused on the microbial communities that inhabit the gut and oral cavity. Mounting evidence suggests that microbial DNA can be detected in the blood circulation using a range of molecular methods. This DNA may represent an untapped pool of biomarkers that have the potential to report on changes to the microbiome of distant sites (e.g., example, the gut and oral cavity). To this end, through amplification and sequencing of the bacterial 16S rRNA variable region four, we evaluated the presence and identity of microbial DNA in blood samples obtained from RA patients (both prior to and 3 months following the instigation of treatment) in comparison to a small number of healthy control subjects and samples obtained from patients with ankylosing spondylitis (AS) and psoriatic arthritis (PA). Bacterial-derived DNA was identified in the majority of our patient samples. Taxonomic classification revealed that the microbiome community in RA was distinct from AS, PA, and the healthy state. Through analysis of paired patient samples obtained prior to and 3 months following treatment (V0 vs. V3), we found the microbiome to be modulated by treatment, and in many cases, this shift reduced the distance between these samples and the healthy control samples, suggesting a partial normalization following treatment in some patients. This effect was especially evident in seronegative arthritis patients. Herein, we provide further evidence for the existence of a blood microbiome in health and identify specific taxa modulated in disease and following treatment. These blood-derived signatures may have significant utility as disease biomarkers and suggest this area warrants further investigation.

## Introduction

An increasing body of evidence demonstrates that the composition of the microbiome, the genetic material of all microorganisms (bacteria, fungi, viruses, and protozoa) that reside within and on our body, differs in health and disease, and further, that this shift in composition from the healthy state, termed “dysbiosis,” may occur in a disease specific manner (Tani et al., 1997; Vaahtovuo et al., 2008; Liu et al., 2013, 2016; Ebringer and Rashid, 2014; Costello et al., 2015; Scher et al., 2015, 2016a,b; Zhang et al., 2015; McDonald et al., 2016; Wen et al., 2017; du Teil Espina et al., 2018; Lopez-Oliva et al., 2018; Zhao et al., 2018a,b). Many resident microbiota are opportunistic pathogens and have the potential to induce disease if there is a breach in host defense. Such breaches in resistance enable specific microbiota members to overpopulate their usual place of habitation or to colonize areas of the body that they are usually unable to do so (Pflughoeft and Versalovic, 2012). Recent studies have further revealed that evidence of the microbiome (generally at the nucleic acid level) is detectable in the circulatory system and report that this is the result of microorganisms (or parts thereof) moving from their usual place of habitation such as the gut, oral cavity, respiratory tract, into the blood; a process termed atopobiosis (Potgieter et al., 2015; Whittle et al., 2018; Castillo et al., 2019).

It is almost universally accepted that the circulatory system of healthy humans is sterile based upon our general inability to detect proliferating microorganisms (Potgieter et al., 2015). However, a number of studies report the presence of proliferating organisms in the circulation of apparently healthy subjects (Damgaard et al., 2015; Castillo et al., 2019; Whittle et al., 2019) or the presence of microorganism-associated components such as microbial nucleic acids (DNA and RNA) (Nikkari et al., 2001; Gosiewski et al., 2017; Whittle et al., 2019). Further, culture-positive bacteremia following tooth brushing and the use of oral irrigation devices is well-appreciated (Berger et al., 1974; Maharaj et al., 2012), suggesting that the transient presence of organisms within the circulation is well-tolerated in the healthy host. In addition, there are numerous investigations that report the presence of bacterial 16S rRNA sequences in human blood samples in various disease states (diabetes, cardiovascular disease, atherosclerosis, and Kawasaki disease) (Amar et al., 2011, 2013; Trøseid et al., 2014; Abe et al., 2015). For a comprehensive review of these studies, please refer to work by Castillo et al. (2019).

## Rheumatoid Arthritis, Ankylosing Spondylitis, Psoriatic Arthritis, and Dysbiosis

Mounting evidence suggests that the composition and status of the microbiota have a crucial role in the pathogenesis of chronic autoimmune diseases. Indeed, many studies associate the existence of an imbalanced microbial community inside the human body (commonly in the gastrointestinal tract) with the initiation and progression of these disorders (Vaahtovuo et al., 2008; Costello et al., 2015; Scher et al., 2015, 2016a; Zhang et al., 2015; Wen et al., 2017; Gilis et al., 2018; Langan et al., 2018, 2019). Rheumatoid arthritis (RA) is an autoimmune disorder characterized by chronic inflammation of the synovial joints leading to significant pain, swelling, and disability with increased morbidity and mortality. RA is estimated to affect ~1% of the world population (Gibofsky, 2012) while in the UK it was estimated to affect 0.67% of the population in 2014 (Abhishek et al., 2017). Despite this, the underlying etiology of RA is still poorly understood. Ankylosing spondylitis and Psoriatic arthritis belong to a group of inflammatory diseases known as the spondyloarthritides (Au et al., 2017). Ankylosing spondylitis (AS) is a persistent autoimmune condition distinguished by inflammation of the peripheral joints, the axial skeleton, and ligaments (Wen et al., 2017). Psoriatic arthritis (PA) is a persistent inflammatory joint disorder typically observed in patients with psoriasis. Both spondyloarthritides have been associated with changes in the gut microbiome (Gilis et al., 2018).

A number of studies have investigated chronic inflammatory disease-associated dysbiosis at various body sites using a range of molecular and biochemical approaches. RA has been associated with dysbiosis of the gastrointestinal tract (Taneja, 2014; Zhang et al., 2015; Chen et al., 2016; Liu et al., 2016; Horta-Baas et al., 2017; Wu et al., 2017; du Teil Espina et al., 2018; Picchianti-Diamanti et al., 2018; Wang and Xu, 2019), oral cavity (Zhang et al., 2015; Cheng et al., 2017; Beyer et al., 2018; Lopez-Oliva et al., 2018; Mikuls et al., 2018), lung (Scher et al., 2016b), and synovial fluid (Martinez-Martinez et al., 2009; Reichert et al., 2013; Zhao et al., 2018b) across a range of studies. Moreover, these studies have begun to associate RA, or stages thereof, with the presence or absence of specific bacteria, suggesting that the microbiome may afford a valuable source of novel biomarkers and or novel targets for therapeutic modulation (Brusca et al., 2014; Costello et al., 2015; Zhang et al., 2015; Cheng et al., 2017; Horta-Baas et al., 2017; Jethwa and Abraham, 2017; Wen et al., 2017). A number of studies have applied these methodologies to the study of Ankylosing spondylitis (AS) and Psoriatic arthritis (PA), two other chronic inflammatory diseases, and have to date investigated the gastrointestinal, oral and skin microbiome (Jethwa and Abraham, 2017; Langan et al., 2018; Lewis et al., 2019). Taken as a whole, these studies suggest that RA, AS, and PA may result in (or from) pan-microbiome dysbiosis affecting multiple niches.

Various disease states are associated with blood microbiome dysbiosis (Amar et al., 2013; Sato et al., 2014; Lelouvier et al., 2016; Mangul et al., 2016; Ling et al., 2017), and these changes are likely reflective of dysbiosis at a distant site(s) with well-characterized resident microbial communities. We believe that these nucleic acids have leached from classical microbiome niches into the blood. These may represent novel biomarkers for disease pathogenesis. Despite mounting evidence suggesting a key role for dysbiosis in the initiation and development of RA, no study to date has investigated whether such translocation occurs in these highly prevalent disorders, and further, whether such data are of diagnostic, prognostic, or therapeutic value.

To this end, this preliminary study aimed to: (a) characterize the circulating microbiome of patients with rheumatoid arthritis (RA), ankylosing spondylitis (AS), and psoriatic arthritis (PA), relative to a number of healthy control subjects, and (b) explore specific microbiome signatures associated with RA, AS, and PA diseases that may increase our understanding of disease pathogenesis and or reveal a pool of candidate biomarkers for further development.

## Materials and Methods

### Donor Population

This study investigated the presence of bacterial 16S rRNA in the serum of 20 rheumatoid arthritis patients. Serum samples were collected both prior to (RA V0), and 3 months following (RA V3) the instigation of treatment with disease modifying anti-rheumatoid drugs. Ethical approval was obtained (NREC 16/LO/0957, London Brent Research Ethics Committee, IRAS Project ID 198240), and all patients provided written informed consent. In addition to the RA patient cohort, blood component samples from four ankylosing spondylitis (AS) patients, four psoriatic arthritis (PA) patients, and four healthy control subjects were procured to facilitate preliminary exploration of any differences between the different disease states (Table 1). These samples were obtained from Sera Laboratories Limited. All samples were analyzed anonymously, and the authors obtained ethical approval (Keele University ERP3) and written informed consent to utilize the samples for the research reported herein. All methods were performed in accordance with relevant guidelines and regulations.

**Table 1:** Patient population.

**Patient ID**	**Disease status**	**RF/CCP status**	**Therapy**
RA 135	Rheumatoid	Positive	MTX monotherapy
RA 138	Rheumatoid	Negative	MTX/SSZ/HCQ
RA 109	Rheumatoid	Negative	MTX monotherapy
RA 140	Rheumatoid	Negative	MTX monotherapy
RA 150	Rheumatoid	Negative	MTX/SSZ/HCQ
RA 151	Rheumatoid	Positive	MTX
RA 145	Rheumatoid	Positive	MTX/SSZ/HCQ
RA 139	Rheumatoid	Negative	SSZ/HCQ
RA 146	Rheumatoid	Positive	MTX/HCQ
RA 111	Rheumatoid	Negative	MTX/HCQ
RA 141	Rheumatoid	Positive	MTX/SSZ/HCQ
RA 115	Rheumatoid	Negative	MTX/SSZ/HCQ
RA 143	Rheumatoid	Negative	MTX/SSZ/HCQ
RA 103	Rheumatoid	Positive	MTX/HCQ
RA 113	Rheumatoid	Positive	MTX
RA 128	Rheumatoid	Positive	MTX/SSZ/HCQ
RA 116	Rheumatoid	Negative	MTX/SSZ/HCQ
RA 144	Rheumatoid	Negative	MTX/SSZ/HCQ
RA 112	Rheumatoid	Positive	MTX
RA 107	Rheumatoid	Negative	MTX
BRH1095340	Ankylosing spondyliztis	NA	MTX
BRH1095341	Ankylosing spondylitis	NA	MTX
BRH1095342	Ankylosing spondylitis	NA	Enbrel/Indocin
BRH1095343	Ankylosing spondylitis	NA	MTX/Enbrel
BRH1095350	Psoriatic arthritis	NA	SSZ
BRH1095351	Psoriatic arthritis	NA	Dovonex
BRH1095903	Healthy control	NA	-
BRH1095904	Healthy control	NA	-
BRH1095905	Healthy control	NA	-
BRH1095906	Healthy control	NA	-

*RF, Rheumatoid Factor; CCP, Anti-cyclic citrullinated peptide; MTX, methotrexate; SSZ, sulphasalazine; HCQ, hydroxychloroquine*.

### Microbiome Characterization

Amplification and sequencing of bacterial 16S rRNA variable region 4 were used to characterize the bacterial community members present in the samples. The bacterial 16S rRNA was amplified by PCR utilizing the oligonucleotide primers listed in Table 2. First round PCR utilizing primers 16SV4_F and 16SV4_R was carried out in reactions comprising 4 μl of each serum, 10 μl Phusion blood PCR buffer, 0.4 μl (2 U) Phusion blood DNA polymerase, 1 μl of each primer of (10 uM) 16S rRNA, and 3.6 μl of the nuclease-free water that had been subjected to 15 min UV-irradiation, in a final volume of 20 μl. A negative control reaction in which serum was replaced with an equal volume of UV-irradiated nuclease-free water was included in each experiment to confirm that none of all reagents were contaminated by target DNA. A positive control reaction (*Escherichia coli* genomic DNA as a template) was also prepared to confirm successful PCR amplification in each experiment.

**Table 2:** Oligonucleotide primers used in this study.

**Primer name**	**Primer sequence (5^**′**^–3^**′**^)**	**Length**
16SV4_F	GTGCCAGCMGCCGCGGTAA	19
16SV4_R	GGACTACHVGGGTWTCTAAT	20
16SV4_XT_F	TCGTCGGCAGCGTCAGATGTGTATAAGAGACAGGTGCCAGCMGCCGCGGTAA	52
16SV4_XT_R	GTCTCGTGGGCTCGGAGATGTGTATAAGAGACAGGGACTACHVGGGTWTCTAAT	54

Cycling parameters comprised an initial denaturation step performed at 98°C for 5 min followed by 33 cycles of: denaturation (98°C, 10 s), annealing (55°C, 5 s), and extension (72°C, 15 s). A final elongation of 7 min at 72°C completed the reaction. Following electrophoretic separation of 5 μl of each resulting PCR product to confirm successful amplification, the remainder (15 μl) was purified of excess primer and PCR reagents using the QIAquick PCR Purification kit. A “kit control” was run alongside this process and involved the purification of 15 μl of UV irradiated nuclease-free water, again to ensure the kit used was free from contaminating target DNA.

In order to add sequencing adapters to facilitate MiSeq library preparation, a second round of PCR amplification was conducted in a total volume of 50 μl comprising 10 μl 5 × Platinum Super-Fi Buffer, 1 μl 10 mM dNTP mixture, 0.5 μl Platinum Super-Fi polymerase, 2.5 μl of each XT_tagged primer (16SV4_XT F/R), 5 μl from the successful first round PCR reaction, and 38.5 μl of UV-irradiated nuclease free water. Cycling parameters comprised an initial denaturation step performed at 98°C for 2 min, followed by seven cycles of denaturation (98°C, 10 s), and annealing/extension (72°C, 20 s) extension (72°C, 15 s). A final elongation of 5 min at 72°C completed the reaction. PCR products were purified utilizing AMPure XP magnetic beads (Agencourt) at a ratio of 0.8 beads to sample (v/v), eluted in 20 μl of UV-irradiated nuclease-free water and quantified utilizing the Qubit 3.0 high-sensitivity DNA kit (Invitrogen).

Amplicons were barcoded using the Nextera DNA library kit, multiplexed for efficiency and sequenced using the Ilumina MiSeq system with a 250 bp paired-end read metric. All samples underwent library preparation at the same time and were sequenced on the same sequencer run. Three negative control reactions, subjected to all experimental procedures, were included on the sequencer run. Bioinformatic analysis was performed using QIIME implemented as part of the Nephele 16S paired-end QIIME pipeline using open reference clustering against the SILVA database for bacteria at a sequence identity of 99%. All other parameters remained as default.

The statistical significance of differences in the abundance of individual bacteria among the RA, AS, PA, and control donors was determined by the Kruskal Wallis test. In order to detect differentially abundant bacterial species between pre- and post-treatment RA patients (RA V0 and RA V3), a paired Wilcoxon Signed-Rank test was applied. In all cases, *P* ≤ 0.05 was considered statistically significant following multiple testing correction.

## Results

### 16S rRNA PCR Amplification

Blood samples were obtained from 32 human subjects in total, as described in Table 1. Among these, 20 were from patients with clinician diagnosed RA who provided serum samples on their first visit (V0) and at their 3-months review (V3). In addition, samples from four patients with AS, four patients with PA, and four healthy control subjects were investigated. Of the 20 patients with RA, 11 were negative for Rheumatoid factor (RF) and/or Anti-cyclic citrullinated peptide (CCP) negative while nine were positive for RF/CCF. All patients with AS and PA recruited for the study were receiving treatment for their respective condition.

Bacterial 16S rRNA amplification provided a total of 17 paired RA samples for analysis. Bacterial 16S rRNA amplification was successful in 100% (4/4) AS, 50% (2/4) PA patients, and 100% (4/4) healthy donors providing a further 10 samples for comparison. Our numerous experimental controls (free template/kit controls) failed to generate a visible band following PCR amplification and gel electrophoresis. Absence of amplified products was subsequently confirmed by analysis with the QuBit high-sensitivity DNA analysis system (ThermoFisher). As a further precaution, we reviewed additional negative control reactions that were sequenced on the same sequencing run and at the same time as the samples reported herein. One such sample generated mappable sequencing data (sample NEGF) and comprised a small number of reads mapping to *Halomonas (6 reads), Corynebacterium 1 (64), Staphylococcus (24), Ralstonia (1,726), Stenotrophomonas (460), Pseudomonas (276), Escherichia*-*Shigella (2,420)*, and *Ruminococcus* (405) but was overwhelmingly composed of reads mapping to the genus *Serratia (18,000)*. In order to control for this potential source of contamination, we highlight any taxa that were found within sample NEGF at a level >25% of the mean experimental sample level i.e., on average, we require a potential contaminant to be observed at a level greater or equal to four times that observed in the negative control to consider its presence as convincing. Application of this approach identified *Serratia, Escherichia-Shigella, Ralstonia*, and *Ruminococcus* as potential contaminants, and discussion of these taxa will make reference to this fact.

### Characterization of the Circulating Bacterial Community *via* 16S rRNA Sequencing

**Figure 1:**
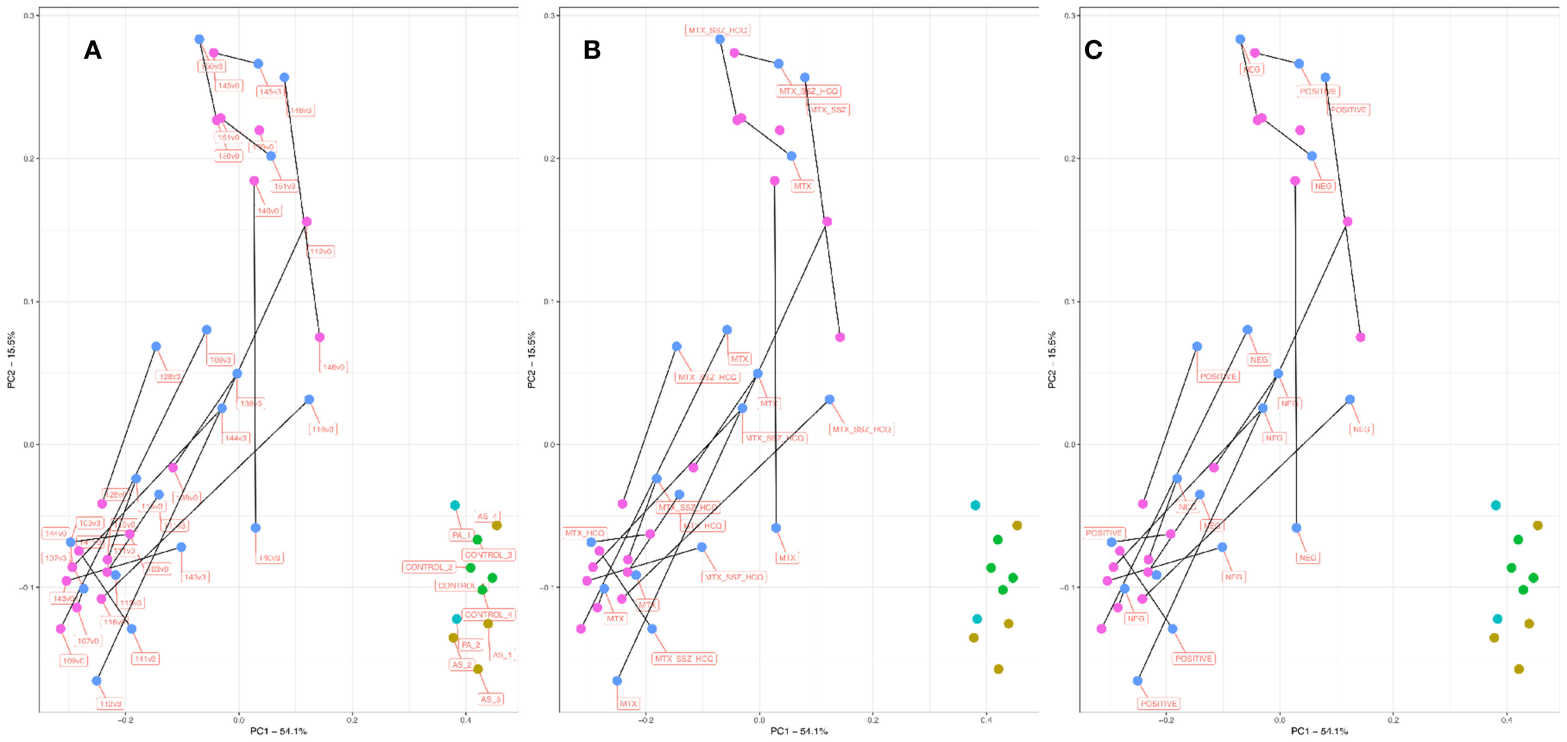
PCoA plot informed by weighted unifrac distance matrix for RA V0 (pink), RA V3 (blue), AS (aqua), PA (mustard), and control subjects (green). Distance matrix informed by amplification and sequencing of the 16S rRNA variable region four followed by taxonomic assignment. Where applicable, paired samples (V0, V3) are linked by means of a black line. Proportions of variation explained by principal coordinates one and two are designated on the relevant axes. Variation explained by the PCoA axes of ordination was 53.3% (PC1) and 15.22 % (PC2). **(A)** Samples are labeled by unique sample ID. **(B)** Samples are labeled by treatment type. **(C)** Samples are labeled by RF/CCP status. RF, Rheumatoid Factor; CCP, Anti-cyclic citrullinated peptide.

The existence and characterization of microbial DNA in the serum were assessed by way of PCR amplification and sequencing of the bacterial 16S rRNA gene (variable region 4) followed by bioinformatic analysis using QIIME (see *Methods*). Our primary approach used principal coordinates analysis (PCoA) to reduce the complexity of the data obtained and to immediately visualize any obvious clustering among samples of different treatment types (Figures 1A–C). Following ordination, it was immediately obvious that the samples from our RA cohort clustered separately to those from the PA, AS, and control patient samples. Indeed, the RA samples clustered markedly further away from the control samples than did the PA or AS samples. These data suggest that the bacterial community composition detected in the blood of our RA patients was more markedly different from that of control (Figure 1A) than were the blood microbiome communities of the PA or AS patient samples.

Principal Coordinate 1 accounted for >50% of the total variance in the dataset, and samples appeared to cluster along this axis (X) with control samples clustering toward the right, and RA samples clustering toward the left. Given the paired nature of our RA cohort, we linked pre- and post-treatment measurements within in each patient (V0 and V3 shown in **pink** and **blue**, respectively) by means of a black connecting line. For 13/17 sample pairs, the microbiome community appeared to progress along Principal Coordinate 1 following treatment, suggesting that the blood microbiome community in these samples became more similar to that of the healthy control/ PA/AS population following treatment.

In order to explore this effect in more detail, we considered the above ordination in light of treatment type (Figure 1B) and RF/CCP status (Figure 1C). Of the 17 RA patients for which complete paired information was available (a successful microbiome analysis pre- and post-treatment initiation), six patients were prescribed methotrexate monotherapy (MTX), two patients methotrexate plus hydroxychloroquine (MTX_HCQ), one patient methotrexate plus sulphasalazine (MTX_SSZ), and eight patients received a combination of all three drugs (MTX_HCQ_SSZ). The 13 sample pairs that progressed toward the right along PC1 post treatment came from patients treated with a range of drug combinations (MTX, MTX_HCQ, MTX_HCQ_SSZ, and MTX_SSZ). Furthermore, the four patient samples that did not progress in this direction also came from patients treated with a range of approaches (MTX, MTX_HCQ, MTX_HCQ_SSZ, and MTX_SSZ), suggesting that the observed shift in microbiome community membership was not influenced by treatment modality. Conversely, of the 13 patients that progressed toward the right along PC1 post treatment, 10 (77%) patients were RF/CCP negative and just three patients RF/CCP positive. In contrast, three out of the four (75%) patients that did not progress towards the right post treatment were RF/CCP positive. These data suggest a role for RF/CCP status in modulating the microbiome response following the commencement of treatment with those patients with RF/CCP negative RA more likely to progress toward a control/PS/PA microbiome community than those who are RF/CCP positive.

### Microbiome Community Composition

**Figure 2:**
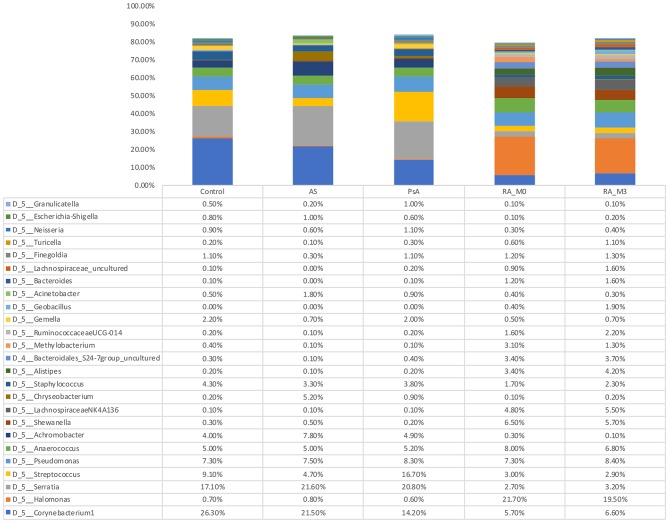
Relative abundance of genera detected within the blood. Data are the relative abundance of the major bacterial taxa, characterized as having a mean abundance of >1% of the total bacteria content in any one experimental group, identified in the serum of rheumatoid arthritis (RA V0, *n* = 18, and RA V3, *n* = 18 or which *n* = 17 are paired), ankylosing spondylitis (AS, *n* = 4), psoriatic arthritis (PA, *n* = 2), and control (Control, *n* = 4) samples as determined using amplification and sequencing of the 16S rRNA gene variable region 4. Data are mean abundance expressed as a percentage of the total bacterial sequence count.

At the phylum level, the circulating microbiome of our study samples was relatively consistent between the different patient groups and predominated by *Proteobacteria* (45.8% of all bacterial DNA) followed by *Firmicutes* (31.40%), *Bacteroidetes* (10.9%), and *Actinobacteria* (10.20%). At the genus level (Figure 2 and Supplementary Data 1), differences between the various patient groups were evident. Control blood samples were predominated by the genera *Corynebacterium 1* (26.3%), Serratia^*^ (17.10%), *Streptococcus* (9.1%), *Pseudomonas* (7.3%), *Anaerococcus* (5.0%), *Staphylococcus* (4.3%), and *Achromobacter* (4.0%). Blood samples from our AS patients contained the same core genera and were predominated by Serratia^*^ (21.6%), *Corynebacterium 1* (21.5%), *Achromobacter* (7.8%), *Pseudomonas* (7.5%), *Anaerococcus* (5.0%), *Streptococcus* (4.7%), and *Staphylococcus* (3.3%). Blood samples from our two PA patients were similar and comprised Serratia^*^ (19.5, 22.60%), *Streptococcus* (22.3, 8.0%), *Corynebacterium 1* (14.3, 14.0%), *Pseudomonas* (7.2, 10.1%), *Anaerococcus* (6.4, 3.3%), *Achromobacter* (2.8, 8.1%), and *Staphylococcus* (3.1, 4.9%). In contrast, our RA samples were more distinct and predominated by *Halomonas* (21.7, 19.5% for V0 and V3, respectively)*, Anaerococcus* (8.0, 6.8%), *Pseudomonas* (7.3, 8.4%), *Corynebacterium 1* (5.7, 6.6%), *Shewanella* (6.5, 5.7%), and members of the *Lachnospiraceae NK4A136 group* (4.8, 5.5%). ^*^Potential contaminant present within a single negative control reaction at a level exceeding that observed in our experimental samples.

**Figure 3:**
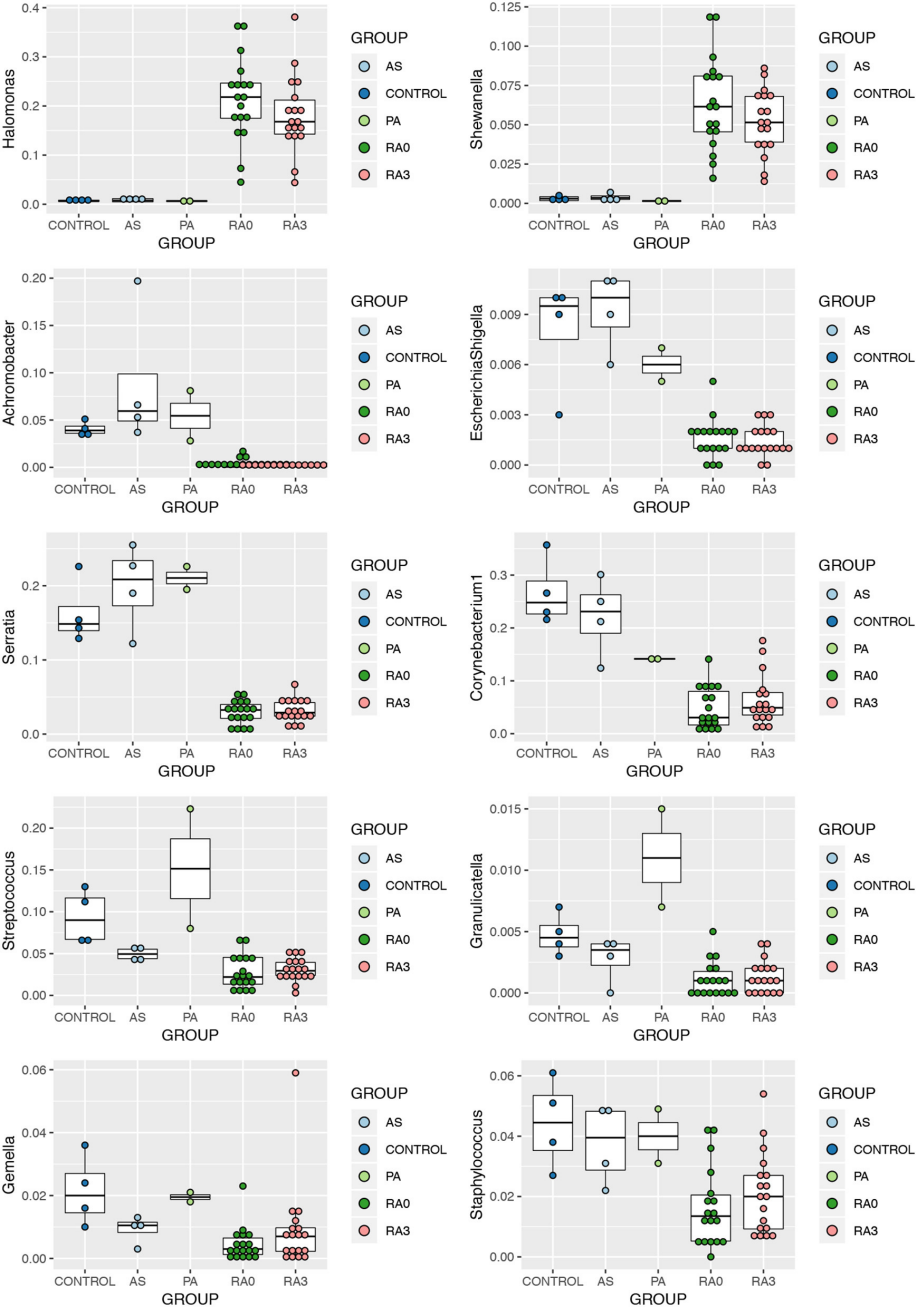
Relative abundance of significantly altered genera detected in the serum of rheumatoid arthritis (RA V0 and RA V3), AS, and PA relative to healthy control serum. Data determined through the amplification and sequencing of the 16S rRNA gene. Data are mean abundance expressed as a percentage of the total bacterial sequence count and scaled between 0 and 1.

Statistical analysis of those genera representing at least 1% of any one experimental group was conducted using the Kruskal Wallis test. Nineteen genera were significantly altered by disease status and are detailed in Table 3. *Post hoc* analysis was performed using the Dunn Test with Benjamini-Hochberg correction for multiple comparisons. At presentation, and prior to treatment (V0), rheumatoid arthritis was associated with significantly elevated levels of *Halomonas* and *Shewanella* and significantly decreased levels of Achromobacter, Escherichia-Shigella^*^, Serratia^*^, Corynebacterium 1, Streptococcus, Granulicatella, Gemella, and *Staphylococcus* relative to our healthy control subjects (Figure 3). Comparison of the means of V0 and V3 revealed no significant changes in these specific genera when the patients provided a further blood sample following 3-months of treatment (V3). Interestingly, differences in the circulating microbiome composition between our healthy control donors and those patients with AS or PA were not statistically significant.

**Table 3:** Genera significantly altered by disease status.

**Taxonomy**	**Kruskal Wallis *P-*Value**	**PA FDR *P*-value**	**AS FDR *P-*value**	**RA V0 FDR *P-*value**	**RA V3 FDR *P-*Value**
D_5__*Halomonas*	0.000	*ns*	*ns*	0.006	0.02
D_5__*Shewanella*	0.000	*ns*	*ns*	0.009	0.01
D_5__*Achromobacter*	0.000	*ns*	*ns*	0.010	0.008
D_5__Escherichia-Shigella^*^	0.000	*ns*	*ns*	0.007	0.005
D_5__Serratia^*^	0.000	*ns*	*ns*	0.009	0.01
D_5__*Corynebacterium 1*	0.000	*ns*	*ns*	0.004	0.007
D_5__*Streptococcus*	0.001	*ns*	*ns*	0.007	0.01
D_5__*Granulicatella*	0.003	*ns*	*ns*	0.03	0.02
D_5__*Gemella*	0.004	*ns*	*ns*	0.01	0.05
D_5__*Lachnospiraceae NK4A136 group*	0.005	*ns*	*ns*	0.07	0.05
D_5__*Staphylococcus*	0.005	*ns*	*ns*	0.03	*ns*
D_5__*Chryseobacterium*	0.005	*ns*	*ns*	*ns*	*ns*
D_5__*Acinetobacter*	0.010	*ns*	*ns*	*ns*	*ns*
D_5__*Ruminococcaceae* UCG-014	0.010	*ns*	*ns*	*ns*	*ns*
D_5__*Neisseria*	0.013	*ns*	*ns*	*ns*	*ns*
D_5__*Turicella*	0.018	*ns*	*ns*	*ns*	*ns*
D_5__*Alistipes*	0.035	*ns*	*ns*	*ns*	*ns*
D_5__*Methylobacterium*	0.043	*ns*	*ns*	*ns*	*ns*
D_5__*Bacteroides*	0.044	*ns*	*ns*	*ns*	*ns*

* *Taxa previously associated with contamination and identified within a single negative control reaction run at the same time as the samples reported herein. ns, not statistically significant*.

**Figure 4:**
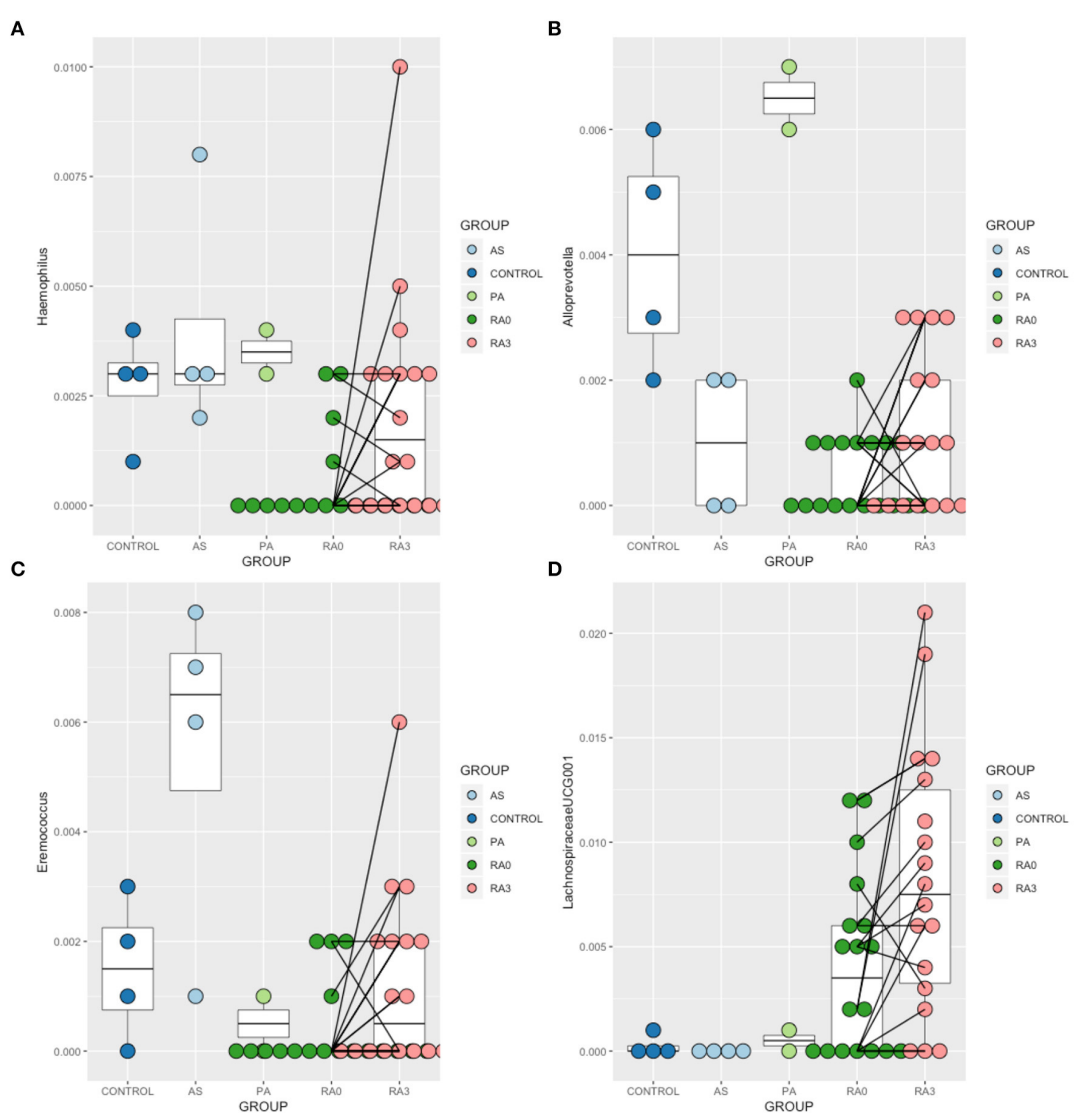
Relative abundance of those taxa identified as being treatment-responsive **(A)** *Haemophilus*, **(B)** *Alloprevotella*, **(C)** *Eremococcus*, **(D)** *Lachnospiraceae*_UGC-001. Data are mean abundance expressed as a percentage of the total bacterial sequence count and scaled between 0 and 1. Black lines link paired observations.

In order to further explore the results of our ordination which suggested that the circulating microbiome of many of our RA patients moved closer toward the healthy control state post treatment, we analyzed the V0 and V3 data for all taxa taking into account the paired nature of these observations. Wilcoxon Signed-Rank Test analysis revealed that members of the genera *Haemophilus, Alloprevotella, Eremococcus*, and *Lachnospiraceae*_UCG001 were significantly altered between V0 and V3 (Figure 4).

## Discussion

There is emerging evidence to support the hypothesis that pan-microbiome dysbiosis in the gut and other locations of patients with RA, AS, and PA is associated with the pathogenesis of disease or disease status (Gilis et al., 2018). However, changes in the blood microbiome of patients with RA, AS, and PA remain understudied. The presence of microbial DNA, either within bacterial cells or in free-form, creating a circulating microbiome is a topic that is being still debated by some (Castillo et al., 2019). However, recent studies have provided evidence for the existence of circulating microbial DNA using a range of different methods (Whittle et al., 2019). While skepticism remains about the origin of the bacterial DNA found in these studies (Hornung et al., 2019), we remain confident in the existence of such given the continued observation that such communities differ by disease state, even when samples are completely randomized prior to and during processing.

Our analyses revealed the presence of a complex serum microbiome community in health and disease. At the phylum level, the blood microbiome was predominated by key four phyla; Proteobacteria, *Firmicutes, Bacteroidetes*, and *Actinobacteria*, and these data further support the notion of a core blood microbiome (Amar et al., 2013; Lelouvier et al., 2016; Païssé et al., 2016; Olde Loohuis et al., 2018; Whittle et al., 2018). Many of the genera identified in this study have previously been identified by us in the blood of healthy human donors as part of a separate study, albeit in differing proportions (Whittle et al., 2019), and by others among both healthy and diseased individuals (Damgaard et al., 2015; Lelouvier et al., 2016; Païssé et al., 2016; Santiago et al., 2016; Li et al., 2018; Qiu et al., 2019).

### Microbiome Composition and Disease

Comparison of the serum microbiome of the 17 paired patient samples with RA, four patients with AS, two patients with PA, and the four healthy controls through PCoA revealed that the composition of microbiome of the RA cohort was different from that of AS, PA cohorts, and the healthy controls, the latter three groups being more similar in their composition. AS and PA both belong to the group of conditions termed spondyloarthritides and share more similarities in their pathogenesis than does RA (Lories and Baeten, 2009), supporting their close proximity in PcOA space. All patients with AS and PA recruited for the present study were receiving treatment, though details of their disease activity at the time of donation were unknown. Therefore, either a lack of active disease or a difference in the pathogenesis may be the reasons for the similarity of serum microbiomes among the healthy controls and patients with AS and PA.

Our PcOA analysis identified key differences between the serum microbiome of our patients with RA compared to those with AS, PA, and our healthy control subjects demonstrated by the fact that these samples occupied a distinct location in the PcOA space. The genera *Halomonas, Anaerococcus, Pseudomonas, Corynebacterium, Shewanella, and Lachnospiraceae NK4A136_group* were most abundant in the RA serum. Many of these have also been identified in the human blood microbiome previously with the exception of *Halomonas* species, which accounted for the majority of reads at genus level (Damgaard et al., 2015; Lelouvier et al., 2016; Païssé et al., 2016; Santiago et al., 2016; Li et al., 2018; Qiu et al., 2019).

Following statistical analysis, a number of taxa were found to significantly differ in their abundance between the RA patients and the other three patient groups.

*Halomonas* and *Shewanella* were found to be significantly higher among our RA patients in comparison to PA, AS, or healthy control subjects. *Halomonas* species are a group of halophilic and halotolerant bacteria that have been recently implicated in a limited number of human infections including dialysis associated sepsis (Kim et al., 2013). *Halomonas* species have been identified in the skin microbiome of humans (Grice et al., 2008). Evidence of this genus has also been found in a salt tolerance locus of the human gut microbiome (Culligan et al., 2013) and identified at elevated levels in the gut mucosal microbiome of patients with rectal cancers along with *Shewanella* species. Identification of *Halomonas* in the salivary microbiome has been associated with inflammatory markers such as IL-1β (Acharya et al., 2017), key inflammatory cytokines also associated with RA. However, *Halomonas* has been identified as a possible contaminant due to its presence in negative control samples subjected to sequencing (Santiago et al., 2016; Whittle et al., 2019). That said, only six reads mapping to the genus *Halomonas* were identified in the single negative control returning sequence data relative to a mean of 3,500 reads identified in our experimental samples.

*Shewanella* species have been increasingly associated with human infections (Yousfi et al., 2017). *Shewanella* species have been found to be perturbed in the human gut microbiome in different physiological and disease conditions (Rojo et al., 2017; Smid et al., 2018), and *Shewanella* has been identified in the blood microbiome of healthy people, particularly associated with RBCs (Païssé et al., 2016). Of interest is the fact that both genera found to be increased in patients with RA are salt tolerant. Liao et al. have identified both *Shewanella* and *Halomonas* along with other bacteria to be increased in the gut microbiome of mice fed with chondroitin sulfate. This has been associated with gut inflammation as well as joint inflammation, leading to the hypothesis that the change in the gut microbiome contributes to the pro-inflammatory state (Liao et al., 2017). Despite other studies identifying this genus as a potential contaminant, we found no evidence of such in our negative control reactions. As a number of studies have identified these two genera to co-exist, our findings cannot be disregarded as incidental or contaminants. Animal studies or cell line studies with co-inoculation of these genera would help to explore this lead further.

Achromobacter, Escherichia/Shigella^*^, Serratia^*^, *Corynebacterium-1, Streptococcus, Granulicatella, Staphylococcus*, and *Gemella* were the genera significantly reduced in abundance in our RA cohort. Of these, *Achromobacter* and to a lesser extent Serratia^*^ have been identified as lymphoid tissue resident commensal (LRC) bacteria in humans and animals. A healthy community of LRC bacteria regulate their own growth through IL-22 to regulate systemic inflammation (Fung et al., 2016). Therefore, a reduction in these bacterial DNA in the serum of patients with RA may indicate a pro-inflammatory state. *Gemella* and *Granulicatella* were also found to be reduced in the sub gingival microbiome of patients with RA (Lopez-Oliva et al., 2018), confirming their existence in classical microbiome niches. Members of the genera *Streptococci, Staphylococci*, and *Corynebacterium* are part of the normal microbiome of the skin, oral cavity, as well as the gut. Therefore, their reduction, as we have noted in the serum microbiome of our patients, along with the other changes noted above, hints toward dysbiosis at these distant sites, reflected in the abundance of DNA reaching the circulatory system.

### Effect of Treatment on the Microbiome

A key finding in our study was that the serum microbiome of 13/17 patients with RA progressed along PC1 following the instigation of treatment toward the healthy control/PA/AS samples. Existing evidence reports that the microbiome of different sites does partially normalize with treatment in patients with rheumatoid arthritis (Zhang et al., 2015); however, these findings have not been uniform (Beyer et al., 2018; Mikuls et al., 2018), perhaps due to the different study designs, sampling sites, or the technologies used. While the effect in our present study was not associated with any one specific treatment modality, 3/4 of the patients with a “non-responsive serum microbiome” had seropositive rheumatoid arthritis, while the majority (77%) of those who responded were seronegative. Clearly, further exploration is warranted here. Previous studies have demonstrated a change in the oral and gut microbiome in patients with RA when treated with MTX and Etanercept (Zhang et al., 2015; Picchianti-Diamanti et al., 2018). While many studies do not differentiate between treatment response and seropositivity, a recent study has further demonstrated that seronegative rheumatoid arthritis may show a better response to treatment (Choi and Lee, 2018). In our study, there was a better normalization of the serum microbiome among the seronegative arthritis group, in line with previously published findings. A recent review illustrates the importance of the gut microbiome in drug metabolism, and growing evidence suggests that differences in the gut microbiome community may explain individual drug responses (Zimmermann et al., 2019).

Abundance of the genera *Haemophilus, Alloprevotella, Eremococcus*, and *Lachnospiraceae*_UGC-001 increased significantly with treatment and contributed to the normalization of the microbiome. *Haemophilus* species have been recognized to be depleted in the gut and oral microbiomes of patients with RA (Zhang et al., 2015; Wu et al., 2016), and our blood-based characterization supports this. *Lachnospiraceae*_UGC-001 was found to be increased with disease and further elevated following treatment. Previous human as well as animal studies have further found these taxa to be increased in individuals with RA (Liu et al., 2016; Wu et al., 2016). *Alloprevotella* species were previously found to be increased in the oral microbiome of patients with RA irrespective of the status of periodontal hygiene (Wolff et al., 2017), whereas we found this genus to be decreased in RA relative to the healthy state. No studies consider *Eremococcus* in relation to RA to date. While our findings were not in full agreement with enriched/depleted taxa found at different body sites in RA in previous studies, one must remember that our analyses are conducted in the blood which likely receives microbial DNA from a range of niches. On this basis, these results certainly warrant further investigation.

### Limitations

It is noteworthy here that all samples were processed in parallel and in a randomized order to avoid the introduction of batch effects. In exploring the fact that our RA cohort clustered entirely separately from the other samples in our PcOA analysis, it is imperative to highlight here that while we closely controlled all analytical parameters from the moment of sample acquisition, our sample cohorts were procured from different sources, arriving as blood product samples in sterile plasticware. It is possible therefore that some differences in their microbiome composition could be explained by variables for which data were not available for our procured material, differences in collection technique or even the immediate environment at the time of collection. We are aware that the microbiome is modulated by a range of factors, and such analyses are highly sensitive to pre-analytical conditions. While we were unable to practically address this limitation herein given the retrospective nature of our study design, we advocate strongly for further prospective studies in this area, which control pre-analytical conditions inclusive of the sample collection phase.

## Concluding Remarks

Through amplification and sequencing of the bacterial 16S rRNA gene, we describe the existence of a blood microbiome in health and in disease. We identify specific taxa that appear to correlate with disease and a further distinct set of taxa that appear to correlate with treatment response. Community level analysis (informed by PcOA) demonstrates that the microbiome of our RA patients appeared to change following treatment. In the main, the taxa identified as disease or treatment responsive have been previously described as common inhabitants of the human microbiome, more commonly found in the oral cavity and gut. These data support our developing hypothesis that microbial DNA found within the blood translocates from more classical microbiome niches that are undergoing disease-association perturbation and therefore, has the potential to serve as a novel biomarker in RA pathogenesis and treatment response. Further studies are required to investigate these preliminary findings.

## Data Availability Statement

The raw data supporting the conclusions of this manuscript will be made available by the authors, without undue reservation, to any qualified researcher.

## Ethics Statement

The studies involving human participants were reviewed and approved by Ethical approval was obtained (NREC 16/LO/0957, London Brent Research Ethics Committee, IRAS Project ID 198240) and all patients provided written informed consent. The patients/participants provided their written informed consent to participate in this study.

## Author Contributions

DH, SH and DT conceived the original study. DH and DT analyzed the sequencing data and produced the final figures. DH, DT, VL, and SH interpreted the data and contributed toward writing of this manuscript. All authors approved the final manuscript.

### Conflict of Interest

The authors declare that the research was conducted in the absence of any commercial or financial relationships that could be construed as a potential conflict of interest.
